# Association of circadian rhythm with mild cognitive impairment among male pneumoconiosis workers in Hong Kong: a cross-sectional study

**DOI:** 10.1038/s41598-023-28832-5

**Published:** 2023-01-30

**Authors:** Bixia Huang, Gengze Liao, Priscilla Ming Yi Lee, Chi Kuen Chan, Lai-bun Tai, Chun Yuk Jason Tsang, Chi Chiu Leung, Lap Ah Tse

**Affiliations:** 1 JC School of Public Health and Primary Care, The Chinese University of Hong Kong, Hong Kong SAR, China; 2 Pneumoconiosis Clinic, Tuberculosis and Chest Service, Department of Health, Hong Kong SAR, China; 3 Pneumoconiosis Mutual Aid Association, Hong Kong SAR, China; 4 Stanley Ho Centre for Emerging Infectious Diseases, The Chinese University of Hong Kong, Hong Kong SAR, China

**Keywords:** Diseases, Health care, Health occupations, Medical research, Risk factors, Signs and symptoms

## Abstract

Weakened circadian activity rhythms (CARs) were associated with mild cognitive impairment (MCI) in the general population. However, it remains unclear among pneumoconiosis patients. We aimed to address this knowledge gap. This cross-sectional study comprised 186 male pneumoconiosis patients (71.3 ± 7.8 years) and 208 healthy community men. Actigraphy was used to determine CARs parameters (percent rhythm, amplitude, MESOR, and acrophase). Values below the corresponding medians of the CARs parameters represented weakened CARs. The Cantonese version of Mini-Mental State Examination (CMMSE) was used to assess cognitive function, MCI, and the composite outcome of MCI plus cognitive impairment. Compared with the community referents, pneumoconiosis patients had worse cognition and dampened CARs. Compared with the community referents or pneumoconiosis patients with robust circadian rhythm, pneumoconiosis patients with weakened circadian rhythm were consistently associated with increased risk of MCI and the composite outcome. However, significant association was only observed between MESOR and the composite outcome (adjusted OR = 1.99, 95%: 1.04–3.81). A delayed phase of CARs was insignificantly associated with MCI and the composite outcome. Our findings showed that weakened CARs were associated with worse cognitive function among male pneumoconiosis workers. Intervention in improving CARs may mitigate cognitive deterioration in male pneumoconiosis workers.

## Introduction

Pneumoconiosis is the most common interstitial occupational lung disease, mainly including silicosis, asbestosis, and coal workers' pneumoconiosis^1^. Globally, 251,299 workers died from pneumoconiosis in 1990, and the death toll slightly rose to 259,700 in 2013^2^. In Hong Kong, silicosis has been ranked as the top third occupational disease, which along with asbestosis, has constantly contributed to 19.4% of overall prescribed occupational diseases over the last decade (2009 ~ 2019)^3^. The development of pulmonary fibrosis could continue even after the dust exposure has been ceased for many years. Some pneumoconiosis workers may suffer from hypoxemia—inflammation, oxidative stress—brain parenchymal, and vascular changes^4^. Evidence from the general older population and patients with chronic obstructive pulmonary diseases (COPDs) has shown that these pathological changes were associated with the development of hippocampal atrophy and an elevated level of amyloid-β protein in the brain^5,6^, which are evident markers of cognitive decline and cognitive impairment^7^.

Meanwhile, as pneumoconiosis is a typical restrictive lung disease, pneumoconiosis workers may also encounter sleep disturbance and poor sleep quality resulting from the related nocturnal cough and breathing difficulties. The sleep disruption may cause pneumoconiosis patients to exposure to more light at night and physical inactivity, disrupting their circadian rhythm. Circadian rhythm is crucial for mammals to maintain synchrony between internal physiology, behavior, and external fluctuating environment^8^. The loss of this synchrony might cause circadian misalignment and further lead to a series of adverse health outcomes, including cardiometabolic diseases^9^, inflammatory diseases^10^, cancer^11^, and neurodegenerative diseases^12^. Recent research in the general aged population has raised great interest in a positive association between circadian rhythm disruption and cognitive impairment^13–15^. Interventions targeted at improving circadian rhythms, such as light therapy^16^, melatonin supplement^17^, and promotion of physical exercise^18^, have shown potential beneficial effects on preventing cognitive decline.

Pneumoconiosis workers are hypothesized to be more vulnerable to weakened circadian rhythm and impaired cognitive function. However, no study has investigated the rest-activity circadian rhythm pattern in pneumoconiosis workers, and the association of circadian rhythm with the cognitive function in pneumoconiosis workers remains unknown. This study aimed to characterize circadian rhythm among pneumoconiosis workers and its association with mild cognitive impairment (MCI) and cognitive impairment, using community people as the reference.

## Methods

### Study design and subject recruitment

In Hong Kong, according to the Pneumoconiosis and Mesothelioma (Compensation) Ordinance^19^, "pneumoconiosis" refers to "fibrosis of the lungs due to dust of free silica or asbestos, or dust containing free silica or asbestos, whether or not such disease is accompanied by tuberculosis of the lungs or any other disease of the pulmonary or respiratory organs caused by exposure to such dust". A medical examination is required for diagnosing pneumoconiosis (profusion category 1/0 or higher). The Pneumoconiosis Medical Board determined the diagnosis of pneumoconiosis following the criteria of the International Labor Organization, including silicosis and asbestosis^20^. Workers who are Hong Kong residents aged ≥ 30 years and currently employed in the construction or renovation industry for at least one year are eligible to claim compensation from the Pneumoconiosis Compensation Fund Board.

This study was a sub-study incorporated in a survey aiming to examine the relationship between sleep quality and cognitive function in pneumoconiosis workers. The original survey enrolled pneumoconiosis workers during the annual interviews organized by the Pneumoconiosis Compensation Fund Board in 2018 and 2019. All 1456 workers in the medical surveillance program were invited. As most participants who agreed to participate were men (workers recruited in the survey, men/women: 767/20), we restricted our study population to male workers to reduce the study population's heterogeneity and improve the study power.

Community referents were recruited in two periods of the COVID-19 outbreak in Hong Kong, that is, before the COVID-19 outbreak (2 July 2019–8 January 2020) and between the 2nd and 3rd waves of COVID-19 (23 June 2020–9 July 2020). They were recruited through poster advertisements in collaboration with five non-governmental organizations and seven district council members located in different areas of Hong Kong, including Kwun Tong, Kowloon City, Tsuen Wan, Sham Shui Po, and Kwai Tsing Districts. To be eligible, community referents ought to be: (1) Hong Kong Chinese male residents aged 60 years old or above (the age distribution was matched on frequency with the pneumoconiosis workers); (2) Cantonese or Mandarin speakers; (3) able to complete the survey independently. We excluded participants with physician-diagnosed mental health disorders or other medical conditions preventing them from completing the survey, such as severe hearing or visual impairment.

From October 2018 to September 2020, a total of 767 male pneumoconiosis patients and 236 community subjects agreed to participate in the original survey. Eleven patients withdrew because of busyness or using oxygen inhalation, resulting in 756 pneumoconiosis patients and 236 community subjects who finished a questionnaire-based interview and had cognitive assessment data. They were all further invited to the actigraphy assessment. From May 2019 to September 2020, 205 patients and 214 community subjects agreed to wear a GENEActive device for 168 h with a sleep diary. During the assessment, six pneumoconiosis workers withdrew for the following reasons: allergy (2), hospitalization (1), busyness (1), and watches not returned (2); and one community subject had not returned the watch. After further excluding 7 participants with incomplete data of the Cantonese version of Mini-Mental State Examination (CMMSE) (4 patients and 3 community subjects) and 11 participants with incomplete data on actigraphy assessment (recorded < 120 h) (9 patients and 2 community subjects), an overall 186 pneumoconiosis workers and 208 community subjects were included in the final analysis. The response rate, effective response rate, and completion rate were 27.1%, 24.6%, and 90.7% for pneumoconiosis workers and 90.7%, 88.1%, and 97.2% for community subjects, respectively. The detailed subject recruitment framework is shown in Supplementary Figure S1. Ethics approval for this study was obtained from the Joint Chinese University of Hong Kong—New Territories East Cluster Clinical Research Ethics Committees (CRE-2018.626). Written informed consent was obtained from each participant before the interview was conducted. All methods were performed following the Declarations of Helsinki. This report follows the Strengthening the Reporting of Observational Studies in Epidemiology (STROBE) reporting guideline for cross-sectional studies.

### Data collection and variable specification

Trained researchers conducted a face-to-face interview with every participant using a standardized questionnaire containing information on socio-demographics, lifestyle habits, medical history, sleep medication use, family history of dementia, depression and anxiety, physical activity, and sleep quality. Age was categorized as < 65, 65–74, or ≥ 75 years. Education attainment was categorized as 0–3, 4–6, or > 6 years. Marital status was classified as single/divorced/widowed or married/cohabitating. Employment status was categorized as retired or employed. Smoking status was categorized as never smoker, former smoker, and current smoker. A never smoker referred to one who had never smoked as much as 20 packs of cigarettes or 12 oz of tobacco in a lifetime, or 1 cigarette a day or 1 cigar a week for 1 year. If a smoker had quit smoking for 1 year or more, he was considered a former smoker 21; otherwise, he was considered a current smoker. Alcohol drinking was classified as never drinker, former drinker, and current drinker. A never drinker referred to one who had never drunk as much as once per month and had been lasting over half a year. A drinker was defined if he or she drank alcohol at least once per month and had been lasting over half a year. If the drinker had quit drinking for 1 year or more, he was considered a former drinker; otherwise, he was a current drinker. Participants who drank tea or coffee more than twice weekly for at least 6 months were defined as tea drinkers or coffee drinkers, respectively. Anxious and depressive symptoms were assessed by the Hospital Anxiety and Depression Scale (HADS)^22^. Both anxiety and depression were categorized as normal (0–7), borderline abnormal (8–10), and abnormal (11–21). Physical activity was assessed with the short interviewer-administrated International Physical Activity Questionnaire (IPAQ) and was categorized as low, moderate, and high^23^. Subjective sleep quality was examined by the Pittsburgh Sleep Quality Index (PSQI), and a poor sleeper was defined if his/her PSQI score was > 5^24^. Waist circumference was measured at the midpoint between the lowest rib and the iliac crest^25^. Handgrip strength was measured by the hydraulic hand dynamometer (Jamar; Lafayette, USA). The maximal handgrip strength measurement from a single trial on either hand was included in the analyses^26^.

### Outcome assessment of mild cognitive impairment and cognitive impairment

Mild cognitive impairment (MCI) is a cognitive decline greater than expected for an individual's age and education level but without notable interference in daily activities^27^. It is a preclinical status between normal cognition to cognitive impairment. The CMMSE was used to measure the cognitive function of the study participants. The CMMSE has been translated and validated by Chiu et al. to assess dementia among Hong Kong Chinese^28^, which contains 30 items to measure various cognitive domains, including orientation, registration, attention and calculation, immediate and short-term recall, and language, with a score ranging from 0 to 30. A lower CMMSE score indicates a worse cognitive function of the participant. We adopted the cut-off levels of CMMSE proposed in a previous study^29^ to define the cognitive status, i.e., 27–30, 21–26, 0–20 were mutually exclusively categorized as normal cognition, MCI, and cognitive impairment (which also means "moderate-severe cognitive impairment"), respectively.

The primary outcome was MCI plus moderate-severe cognitive impairment (i.e., composite outcome). As only a limited number of cognitive impairment cases were obtained, a separate analysis only for MCI was also examined. In the logistic regression model, the dependent variables are binary. If "MCI" = 1, that means a CMMSE score of 21–26; if "composite outcomes of MCI plus cognitive impairment" = 1, that means a CMMSE score of 0–26; both "MCI" = 0 and "composite outcomes of MCI plus cognitive impairment" = 0 refer to a CMMSE score of 27–30, and were used as the reference level.

The Hong Kong version of Montreal Cognitive Assessment (HK-MoCA) was also used to measure the cognitive function of study participants in the sensitivity analysis, which has been validated by Yeung et al.^30^ and Wang et al.^31^. The age and education corrected cutoff scores were adopted to classify the severity of cognitive impairment. A score of > 7th, 7th-2nd, and ≤ 2nd percentile was determined as normal cognition, MCI, and cognitive impairment, respectively^32^. Since no percentile cutoff scores were reported for subjects < 65 years old in the manual, subjects in this age stratum were referred to the percentile scores of the 65–69 age stratum in this study.

### Assessment of circadian activity rhythms

Each pneumoconiosis worker and community subject continuously wore a GENEActiv Original (Activinsights Company, UK) device on his non-dominant wrist for 168 h without removal, even during sleep or bathing (measurement frequency 100 Hz, sampling rate corresponding to 1 min). The assessment of circadian rhythm parameters had been described previously^33^. The actigraphy detects and records movements in three mutually vertical axes (x, y, and z) and real-time skin temperature. A gravity-subtracted sum of vector magnitudes (SVM) was automatically calculated with data of the three axes (x, y, and z) and a formula defined by the manufacturer: SVMg s = [(x^2^ + y^2^ + z^2^)½—1 g]^34^. Non-wearing time was determined by reviewing the activity records outputted from the GENEActiv software and self-reported by the interviewees. The non-wearing periods should present low and steady SVM readings. For each participant, the data of non-wearing periods were excluded from the calculation of their parameters. The recordings lasted from 5 to 7 consecutive days, including a weekend. If the sum length of wearing was less than 120 h (5/7 of 168 h), the wearing was considered incomplete, and its data were not analyzed.

Data of SVM were then imported into the Chronos-Fit program (v. 1.06), in which a cosine curve with a period at or near 24 h fits to the data by the least-squares method. Four circadian rhythm parameters were computed, namely percent rhythm, amplitude (peak-to-nadir difference), the midline estimating statistic of rhythm (MESOR) (mean of the fitted curve), and acrophase (time of peak activity)^35^. Both the amplitude and MESOR reflect the magnitude of a cycle. Phase (advanced or delayed) is defined as the timing of a reference point in the cycle for a certain event. A phase advance means the reference point moves earlier, while a phase delay means the reference point moves later. There were no available standard cutoff points for classifying the levels of percent rhythm, amplitude, MESOR, and acrophase. Thus, we used medians of the four parameters to dichotomize participants with different exposure levels. Percent rhythm, amplitude, and MOSER below the corresponding medians represented weak circadian rhythms, and the robust rhythm was defined if the actual measurement was equal to or above the median. Overall, the larger value of the above three circadian parameters, the more robust the circadian rhythm. Acrophase was used to assess if the circadian phase was delayed or advanced compared with the median level of the participants.

### Statistical analyses

Differences in the basic characteristics, cognitive function, and circadian rhythm parameters between patients and community subjects were compared using the chi-square test or Fisher's exact test for categorical variables, and t-test or Kruskal–Wallis test for continuous variables, according to the data distribution. We dichotomized all participants using the median levels of the four circadian rhythm parameters to examine the association between weakened circadian rhythm and the prevalence of MCI and composite outcome. To illustrate whether pneumoconiosis workers with weakened circadian rhythm parameters had a higher risk of MCI and composite outcome, we further classified study subjects into four sub-groups according to the status of pneumoconiosis (yes/no) and circadian activity rhythm (above/below median levels), using community subjects with robust circadian activity rhythm parameters as the reference. Unconditional multivariable logistic regression was performed to calculate the adjusted odds ratios (ORs) for the association of being diagnosed with pneumoconiosis and levels of circadian rhythm parameters with the presence of MCI and composite outcome, after adjustment of age (years), education attainment (years), marital status, employment, diabetes, hypertension or cardiovascular diseases, sleep medication use, family history of dementia, smoking, alcohol drinking, tea drinker, coffee drinker, anxiety, depression, physical activity, poor sleep (PSQI > 5), waist circumference, and handgrip strength. The covariates were selected based on the conceptual definition of confounding and referred to previous literature with similar study purposes.

Sensitivity analyses were conducted to examine the robustness of the associations of circadian rhythm with MCI and composite outcome by evaluating MCI and cognitive impairment with HK-MoCA. All analyses were performed using SAS statistical software 9.4 (SAS Institute Inc., Cary, NC) and Stata 15 (StataCorp, College Station, TX, US). All tests were two-sided, and a *P* value < 0.05 was considered statistically significant.

## Results

### Sociodemographic characteristics and prevalence of MCI and cognitive impairment among pneumoconiosis patients

Among 186 pneumoconiosis workers included in this report, 182 were diagnosed with silicosis, and 4 were diagnosed with asbestosis. Overall, the average age of the pneumoconiosis patients was 71.3 (s.d.: 7.8) years, with 77.4% current or former smokers and 82.8% low education attainment (< 6 years). Compared with the community subjects, pneumoconiosis patients were more likely to be lower educated, had lower waist circumference, and with a family history of dementia; however, they had a higher proportion of married/cohabitating, retired, former smokers, former alcohol drinkers or tea drinker and poor sleepers (all *p* < 0.05) (as shown in Table 1).

**Table 1:** Comparisons of characteristics between pneumoconiosis workers and community subjects.

Characteristic	Community referents (N = 208)		Pneumoconiosis workers (N = 186)		*P* value
	**n**	**%**	**n**	**%**	
Age group, years					0.30
< 65	28	13.5	34	18.3	
65–74	101	48.6	92	49.5	
≥ 75	79	38	60	32.3	
Education, years					< 0.001
0–3 years	44	21.2	78	41.9	
4–6 years	68	32.7	76	40.9	
> 6 years	96	46.2	32	17.2	
Marital status					0.002
Single/Divorced/Widowed	37	17.8	14	7.5	
Married/cohabitation	166	79.8	170	91.4	
Unknown	5	2.4	2	1.1	
Employment					0.045
Retired	175	84.1	169	90.9	
Employed	33	15.9	17	9.1	
History of diabetes	51	24.5	33	17.7	0.10
History of hypertension or CVDs	112	53.8	102	54.8	0.84
Current sleep medication user	9	4.3	5	2.7	0.43
Family history of dementia	11	5.3	2	1.1	0.023
Smoking status					< 0.001
Current smoker	39	18.8	22	11.8	
Former smoker	68	32.7	122	65.6	
Never smoker	101	48.6	42	22.6	
Alcohol Drinking					< 0.001
Current drinker	35	16.8	37	19.9	
Former drinker	31	14.9	63	33.9	
Never drinker	142	68.3	86	46.2	
Tea drinker	152	73.1	163	87.6	< 0.001
Coffee drinker	68	32.7	47	25.3	0.11
Anxiety score of HADS					0.60
Normal (0–7)	183	88	159	85.5	
Borderline abnormal (8–10)	18	8.7	15	8.1	
Abnormal (11–21)	7	3.4	10	5.4	
Missing	0	0	2	1.1	
Depression score of HADS					0.15
Normal (0–7)	165	79.3	158	84.9	
Borderline abnormal (8–10)	23	11.1	17	9.1	
Abnormal (11–21)	20	9.6	9	4.8	
Missing	0	0	2	1.1	
Physical activity					0.54
Low	40	19.2	32	17.2	
Moderate	107	51.4	106	57	
High	61	29.3	48	25.8	
Poor sleep quality	109	52.4	141	75.8	< 0.001

	Mean	SD	Mean	SD	
Age, years	72.9	8.0	71.3	7.8	0.058
Waist circumference, cm	89.5	10.1	86.2	9.1	< 0.001
Handgrip strength, kg	27.6	7.8	28.6	8.4	0.21

*CVDs* cardiovascular disease; *HADS* Hospital Anxiety and Depression Scale; *SD* standard deviation.

Table 2 summarizes cognitive function and status of cognitive impairment measured by CMMSE among the pneumoconiosis patients with the comparison of community referents. Pneumoconiosis workers presented a significantly lower global score of 25.7 (s.d.: 3.4) than that of the community referents (26.4, s.d.: 3.4) (*p* = 0.045). The poorer global cognitive function in pneumoconiosis patients was mainly attributed to the poor score of the sub-item "orientation" (*p* = 0.011). In addition, the prevalence of the composite outcome, MCI, and cognitive impairment (50%, 38.7%, and 11.3%) in pneumoconiosis patients measured by CMMSE were significantly higher than those of the community referents (36.5%, 29.3%, and 7.2%).

**Table 2:** Distribution of cognitive function measured by CMMSE in pneumoconiosis patients and community referents.

Variable	Community referents (N = 208)		Pneumoconiosis workers (N = 186)		*P* value
	Mean	SD	Mean	SD	
Global score	26.4	3.4	25.7	3.4	0.045
Component scores					
1. Orientation	9.41	1.27	9.09	1.22	0.011
2. Registration	2.86	0.52	2.87	0.43	0.92
3. Attention and calculation	3.73	1.6	3.65	1.59	0.64
4. Recall	2.12	0.98	2.03	1.01	0.35
5. Language	8.24	1.11	8.04	1.25	0.098

	n	%	n	%	
Status of cognitive impairment					0.024
Normal cognition (27–30)	132	63.5	93	50.0	
Mild cognitive impairment (21–26)	61	29.3	72	38.7	
Cognitive impairment (0–20)	15	7.2	21	11.3	
*Composite outcome (0–26)*^*a*^	*76*	*36.5*	*93*	*50.0*	*0.007*

*CMMSE* Cantonese version of Mini-Mental State Examination; *SD* standard deviation.

^a^Composite outcome refers to the prevalence of MCI plus cognitive impairment.

### The pattern of circadian activity rhythm and association with MCI and the composite outcome.

Table 3 demonstrated that the pneumoconiosis patients had relatively lower values of all circadian activity rhythm parameters than those of the community participants. However, the significant differences were only for MESOR (236.4 vs. 268.2, *p* = 0.008) and acrophase (13.5 vs. 14.1, *p* = 0.029).

**Table 3:** Distribution of circadian rhythm parameters in pneumoconiosis patients and community referents.

Variable	Community referents (N = 208)		Pneumoconiosis workers (N = 186)		*P* value
Percent rhythm	18.1	12.8–23.4	17.7	13.6–23.6	0.98
Amplitude	128.9	86.3–181.5	113.2	79.1–169.9	0.13
MESOR	268.2	213.3–327.5	236.4	194.9–309.5	0.008
Acrophase	14.1	12.5–15.6	13.5	12.2–14.8	0.029

Values were given as median (interquartile range).

*MESOR* midline estimating statistic of rhythm.

Associations of circadian rhythm parameters with cognitive function and prevalence of MCI and composite outcome among pneumoconiosis patients are presented in Table 4. By taking patients with the robust circadian rhythm parameter as the reference, patients with weakened circadian rhythm were associated with increased prevalences of MCI and the composite outcome. However, a marginally significant association was only observed between low MESOR value and the composite outcome.

**Table 4:** Associations between circadian activity rhythm and cognitive function in pneumoconiosis patients.

Circadian parameters	CMMSE total score	Mild cognitive impairment	Composite outcome ^a^
Cases, N	186	72	93
Model 1			
Percent rhythm < 17.7	0.11 (− 0.82, 1.04)	1.71 (0.90, 3.24)*	1.31 (0.72, 2.4)
Amplitude < 113.2	0.00 (− 0.97, 0.96)	1.86 (0.96, 3.59)*	1.50 (0.81, 2.78)
MESOR < 236.4	− 0.99 (− 1.96, − 0.03)**	1.71 (0.89, 3.27)	1.88 (1.01, 3.51)**
Acrophase < 13.5	− 0.61 (− 1.55, 0.34)	0.80 (0.42, 1.52)	1.00 (0.54, 1.83)
Model 2			
Percent rhythm < 17.7	0.40 (− 0.58, 1.37)	1.56 (0.78, 3.11)	1.13 (0.59, 2.17)
Amplitude < 113.2	0.42 (− 0.63, 1.47)	1.63 (0.79, 3.36)	1.29 (0.65, 2.54)
MESOR < 236.4	− 0.90 (− 1.94, 0.14)*	1.56 (0.76, 3.19)	1.80 (0.90, 3.60) *
Acrophase < 13.5	− 0.31 (− 1.31, 0.69)	0.65 (0.32, 1.33)	0.83 (0.42, 1.62)

*MCI* mild cognitive impairment; *CMMSE* Cantonese version of the Mini-Mental State Examination; *MESOR* midline estimating statistic of rhythm.

Model 1, adjusted for age (years) and education (years).

Model 2, model 1 further adjusted for marital status, employment, diabetes, hypertension or cardiovascular diseases, sleep medication use, family history of dementia, smoking, alcohol drinking, tea drinker, coffee drinker, anxiety, depression, physical activity, poor sleep (PSQI > 5), waist circumference, and handgrip strength.

**P < 0.05, *P < *0.1.*

^a^Composite outcome refers to the prevalence of MCI plus cognitive impairment.

To further understand if pneumoconiosis patients with weakened circadian rhythms had a higher prevalence of cognitive impairment, we conducted additional analysis by using community subjects with robust circadian rhythms as a reference. As shown in Table 5, pneumoconiosis patients with a lower level of circadian rhythm parameters (i.e., percent rhythm, amplitude, and MESOR) were consistently related to a higher risk of MCI and composite outcome, despite the significant association was only observed between MESOR and the composite outcome. In addition, a higher risk of MCI was indicated among pneumoconiosis patients with a delayed phase of rest-activity rhythm, though there was not statistically significant. Compared to pneumoconiosis patients, attenuated associations between circadian rhythm parameters and MCI or cognitive impairment were generally found among community referents, except for percent rhythm. The lower percent rhythm (< 17.8) was significantly associated with a lower risk of MCI or composite outcome in community referents.

**Table 5:** Association between circadian rhythm and MCI or cognitive impairment assessed by CMMSE in study population.

	Community referents		Pneumoconiosis patients	
	NC/MCI	AOR (95% CI)	NC/MCI	AOR (95% CI)
Percent rhythm ^a^				
≥ 17.8	61/38	1.00 (ref)	48/28	0.73 (0.36, 1.51)
< 17.8	71/23	0.48 (0.24, 0.95) **	45/44	1.16 (0.58, 2.31)
Amplitude ^a^				
≥ 120.8	77/29	1.00 (ref)	50/28	1.13 (0.54, 2.34)
< 120.8	55/32	1.11 (0.56, 2.21)	43/44	1.62 (0.80, 3.25)
MESOR ^a^				
≥ 254.8	81/32	1.00 (ref)	50/26	1.11 (0.54, 2.27)
< 254.8	51/29	1.23 (0.61, 2.49)	43/46	1.74 (0.88, 3.44)
Acrophase ^a^				
≥ 13.8	78/29	1.00 (ref)	42/38	1.89 (0.92, 3.90) *
< 13.8	54/32	1.05 (0.51, 2.15)	51/34	0.95 (0.45, 2.03)

	NC/Composite outcomes of MCI plus CI	AOR (95% CI)	NC/Composite outcomes of MCI plus CI	AOR (95% CI)
Percent rhythm ^a^				
≥ 17.8	61/45	1.00 (ref)	48/43	0.89 (0.46, 1.76)
< 17.8	71/31	0.52 (0.27, 1.00) **	45/50	1.04 (0.53, 2.05)
Amplitude ^a^				
≥ 120.8	77/33	1.00 (ref)	50/37	1.26 (0.64, 2.51)
< 120.8	55/43	1.25 (0.65, 2.41)	43/56	1.66 (0.84, 3.26)
MESOR ^a^				
≥ 254.8	81/37	1.00 (ref)	50/30	1.09 (0.55, 2.18)
< 254.8	51/39	1.49 (0.76, 2.89)	43/63	1.99 (1.04, 3.81) **
Acrophase ^a^				
≥ 13.8	78/35	1.00 (ref)	42/42	1.65 (0.82, 3.33)
< 13.8	54/41	1.03 (0.52, 2.02)	51/51	1.10 (0.55, 2.23)

^a^Using median as the cutoff point.

*MCI* mild cognitive impairment; *CMMSE* Cantonese version of the Mini-Mental State Examination; *MESOR* midline estimating statistic of rhythm; *OR* odds ratio; *CI*, confidence interval.

^b^adjusted for age (years) and education (years), marital status, employment, diabetes, hypertension or cardiovascular diseases, sleep medication use, family history of dementia, smoking, alcohol drinking, tea drinker, coffee drinker, anxiety, depression, physical activity, poor sleep (PSQI > 5), waist circumference, and handgrip strength.

** P < 0.05, * P < *0.1.*

Sensitivity analyses were conducted using HK-MoCA to measure MCI and cognitive impairment and exclusion of community subjects recruited during COVID-19 outbreaks in Hong Kong (Supplementary Tables S1 and S2). A similar pattern was suggested for the association of circadian rhythm parameters with MCI and composite outcome.

## Discussion

This study is the first to investigate the circadian rhythm pattern and its association with MCI and the composite outcome of MCI plus cognitive impairment among pneumoconiosis workers. We observed significant disturbances in cognition and circadian activity rhythm in patients with pneumoconiosis. Compared with the community referents, pneumoconiosis patients with weakened circadian activity rhythm and delayed circadian/activity phase had higher prevalences of MCI and the composite outcome. Our findings suggest that weakened circadian rhythm in pneumoconiosis workers may be a novel marker of impaired cognition, potentially serving as a therapeutic target for mitigating the increasing health problem related to cognitive impairment.

Previous studies on the rest-activity pattern were mainly derived from patients with neurodegeneration^12^, cardiometabolic diseases^9^, cancer^36^, and mental disorders^37^. Evidence about circadian activity rhythm in respiratory diseases (except for lung cancer) is very scarce. Nunes et al.^38^ compared the circadian rest-activity rhythm between 26 clinically stable COPD patients and 15 controls and found that COPD patients had a decrease in the relative amplitude mean (0.696 ± 0.134 vs. 0.833 ± 0.093) than the controls. Although there was no statistically significant finding in amplitude, our study found a statistically significant lower level of MESOR in pneumoconiosis patients, which provided additional evidence of circadian rhythm disturbances in patients with chronic respiratory diseases.

The association between circadian activity rhythm and cognitive function was revealed in the non-demented older population in both cross-sectional and prospective studies. A study conducted in 2754 men (mean age of 76 years) in the US reported that lower baseline amplitude, robustness (pseudo-F-statistic), and MESOR, as well as advanced acrophase, were associated with more significant cognitive decline over 3.4 years of follow-up^13^. Another study on 1287 women (mean age of 83) found that lower amplitude and robustness and delayed rhythms at baseline were associated with worse cognitive performance^15^. Since sleep and circadian rhythm are tightly coupled, circadian rhythms might affect cognition through sleep-dependent and sleep-independent pathways^39^. Animal and human studies have proposed some potential mechanisms for the circadian-cognition relationship^12^, which could be summarized as 1) alter sleep timing and cause sleep deprivation, thus regulating amyloid-β and Tau dynamics and increase inflammatory and neuronal injury^40,41^; 2) regulate inflammatory activation, the circadian clock is critical for innate immune homeostasis in the brain. Conversely, circadian dysfunction could cause neuroinflammation and exacerbate neuropathology^42^; 3) circadian misalignment may directly regulate protein homeostasis and regulate the blood–brain barrier permeability for clearance of protein aggregates, thus influence protein aggregation^43^; 4) oxidative stress^44^.

In line with the findings in the general population, the results of our multivariate analyses consistently indicated higher prevalences of MCI and the composite outcome of MCI plus cognitive impairment in patients with weakened circadian activity rhythm (lower values of amplitude and MESOR). Our results also suggested a more robust circadian rhythm-cognition relationship in pneumoconiosis than in the general population. Our findings that a lower percent rhythm (< 17.8) was associated with a lower risk of MCI or the composite outcome in community referents but not in pneumoconiosis patients might be explained by the cut-off point, as we designed the cut-off point of pneumoconiosis patients to the community referents. As the circadian rhythm are relatively more robust in community referents than the pneumoconiosis patients, their risk estimate could still be robust even if they were grouped into the lower category of percent rhythm.

This study has several strengths. First, we recruited community subjects as referents using the same protocol within our study to avoid potential heterogeneities when comparing the findings with other studies. Second, the rhythm on weekdays and weekends could be substantially different^45^. The long actigraphy wearing time (120 to 168 h, including weekdays and weekends) made us more accurately capture the circadian patterns of the study population. Third, our study has a good completion rate and compliance, as 94.0% of the participants (394 out of 419 subjects who agreed to wear the device) provided credible actigraphy data after the long device wearing. Finally, to reduce the residual confounding bias, we collected comprehensive data on possible cofounders on demographic characteristics, socioeconomic status, lifestyle factors, and medical history.

This study has several limitations. First, the cross-sectional design of this study may limit us from causal inference. However, this study added value to the scientific literature as evidence of circadian rhythm and the cognitive outcome is very limited in pneumoconiosis patients. Second, sixty community subjects in the study were recruited between June 23 to July 09, 2020, just after the 2nd wave of the COVID-19 outbreak in Hong Kong^46^. The possible physical inactivity due to sustained quarantine and social distancing^47^ may adversely influence participants' circadian rhythm. We performed a sensitivity analysis excluding the community subjects recruited within this period. A significantly decreased amplitude was observed in pneumoconiosis patients compared to that of the community subjects (as shown in Supplementary Table S2). Thus, the overall circadian activity of the community referents may be underestimated. However, we expected this would have biased our findings toward the null. Third, we used medians as cut-offs for each circadian rhythm parameter because there are no standard criteria to define weak and robust circadian rhythm. Meanwhile, the sample size restricted us from further dividing participants into tertiles or quartiles of circadian rhythm parameters to investigate the biological gradient (dose–response) of circadian disruption. According to their self-reported disease history, there were no patients with any lung diseases in our community referents. Thus, we could not compare the major outcomes between community residents with or without other lung diseases. We used the composite outcome of MCI plus cognitive impairment as the primary outcome to improve statistical power. Finally, all our study participants were men, so the generalization to the whole population, including women, could be limited.

## Conclusions

The weakened circadian activity rhythm of male pneumoconiosis workers was positively associated with the prevalence of MCI and composite outcome of MCI plus cognitive impairment. Our study suggests that improving circadian rhythm may mitigate cognitive deterioration in male pneumoconiosis workers.

## Supplementary Information

Supplementary Information.

## Data Availability

The datasets used and/or analysed during the current study are available from the corresponding author on reasonable request**.**
