# Supplementary material for: Association of circadian rhythm with mild cognitive impairment among male pneumoconiosis workers in Hong Kong: a cross-sectional study
Source: Sci Rep. 2023 Jan 30;13:1650. doi: 10.1038/s41598-023-28832-5 (PMC9885413; doi:10.1038/s41598-023-28832-5)
Supplement: Supplementary file 1 — Supplementary Information. [file 41598_2023_28832_MOESM1_ESM.pdf]

Association of circadian rhythm with mild cognitive impairment among male pneumoconiosis workers in Hong Kong: a cross-sectional study

Bixia Huang[1], Gengze Liao[1], Priscilla Ming Yi LEE[1], Chi Kuen Chan[2], Lai-bun Tai[2], Chun Yuk Jason Tsang[3], Chi Chiu Leung, MD[4] and Lap Ah Tse[1]*

Authors' affiliations

1 JC School of Public Health and Primary Care, the Chinese University of Hong Kong, Hong Kong Special Administrative Region, China

2 Pneumoconiosis Clinic, Department of Health, Hong Kong Special Administrative Region, China

3 Pneumoconiosis Mutual Aid Association, Hong Kong Special Administrative Region, China

4 Stanley Ho Centre for Emerging Infectious Diseases, the Chinese University of Hong Kong, Hong Kong SAR, China

***Name and address of corresponding author:**

Dr. Lap Ah Tse, JC School of Public Health and Primary Care, the Chinese University of Hong Kong. 4/F School of Public Health and Primary Care, Prince of Wales Hospital, Sha Tin, N.T., Hong Kong SAR, China. Telephone: 852-2252 8791; Fax: 852-2606 3500; E-mail: shelly@cuhk.edu.hk

Supplementary Material

Supplementary Figure S1.

Profile of the study.

Supplementary Table S1.

Association between circadian rhythm and MCI or cognitive impairment assessed by HK-MoCA in study population.

Supplementary Table S2.

Comparisons of circadian activity rhythm between pneumoconiosis patients and community referents (exclude community subjects recruited during the COVID-19 pandemic)

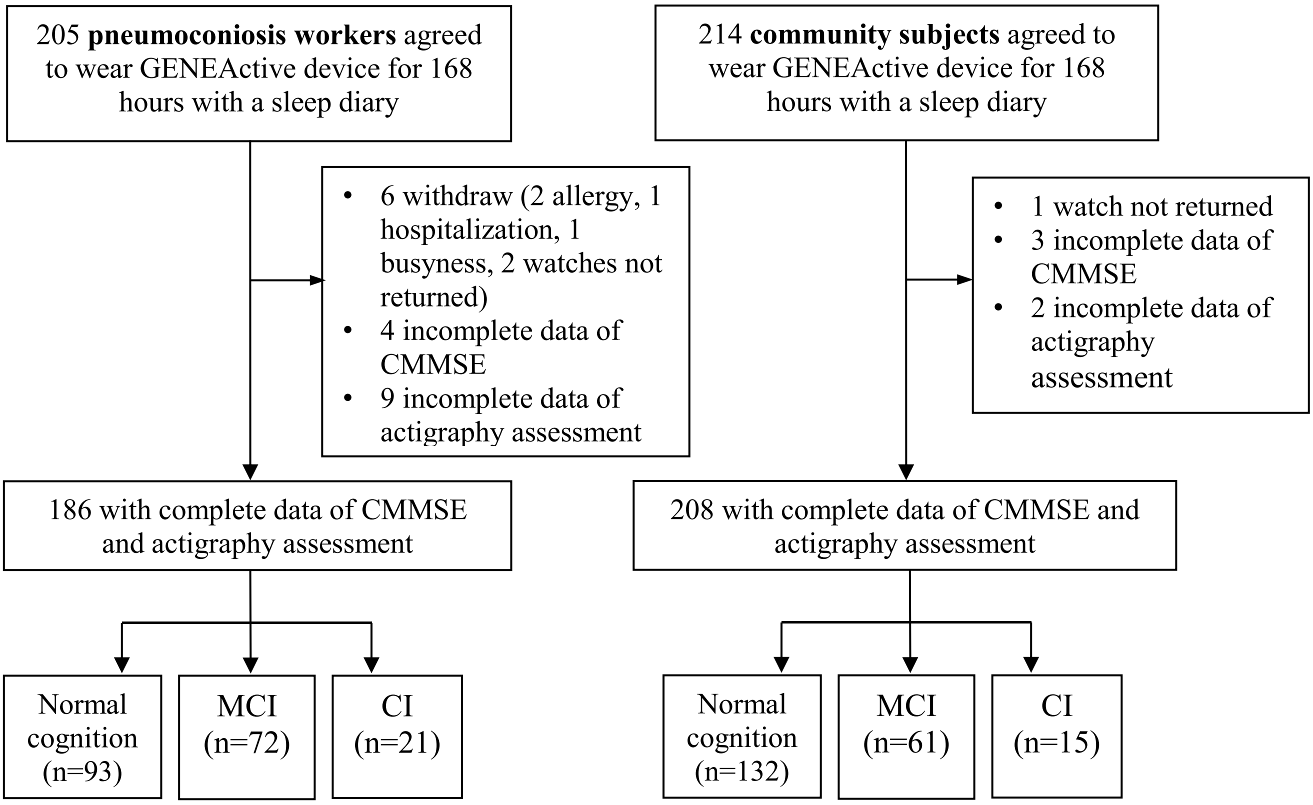

Supplementary Figure S1. Profile of the study

Abbreviation: CMMSE, Cantonese version of the Mini-Mental State Examination; MCI, mild cognitive impairment; CI, cognitive impairment.

Supplementary Table S1. Association between circadian rhythm and MCI or cognitive impairment assessed by HK-MoCA in study population

	**Community referents**		**Pneumoconiosis patients**	
	**NC/MCI**	**AOR (95% CI)**	**NC/MCI**	**AOR (95% CI)**
Percent rhythm [a]				
≥ 17.8	94/7	1.00 (ref)	77/11	1.13 (0.36, 3.56)
< 17.8	89/9	1.35 (0.44, 4.18)	77 /14	1.51 (0.51, 4.51)
Amplitude [a]				
≥ 120.8	98/8	1.00 (ref)	75/8	0.71 (0.22, 2.28)
< 120.8	85/8	1.31 (0.41, 4.17)	79/17	1.97 (0.67, 5.83)
MESOR [a]				
≥ 254.8	105/8	1.00 (ref)	67/10	1.01 (0.32, 3.16)
< 254.8	78/8	1.16 (0.37, 3.65)	87/15	1.36 (0.48, 3.88)
Acrophase [a]				
≥ 13.8	101/7	1.00 (ref)	69/13	1.84 (0.60, 5.67)
< 13.8	82/9	1.37 (0.43, 4.34)	85/12	1.02 (0.32, 3.21)
	**NC/Composite outcomes of MCI plus CI**	**AOR (95% CI)**	**NC/Composite outcomes of MCI plus CI**	**AOR (95% CI)**
Percent rhythm [a]				
≥ 17.8	94/7	1.00 (ref)	77/13	0.96 (0.36, 2.55)
< 17.8	89/9	1.09 (0.43, 2.76)	77 /18	1.33 (0.53, 3.38)
Amplitude [a]				
≥ 120.8	98/11	1.00 (ref)	75/11	0.85 (0.31, 2.32)
< 120.8	85/12	1.26 (0.48, 3.30)	79/20	1.61 (0.63, 4.15)
MESOR [a]				
≥ 254.8	105/11	1.00 (ref)	67/12	1.03 (0.38, 2.83)
< 254.8	78/12	1.24 (0.47, 3.24)	87/19	1.33 (0.53, 3.34)
Acrophase [a]				
≥ 13.8	101/11	1.00 (ref)	69/14	1.43 (0.53, 3.81)
< 13.8	82/12	1.19 (0.45, 3.15)	85/17	1.05 (0.40, 2.78)

[a] Using median as the cutoff point.

Abbreviations: MCI, mild cognitive impairment; HK-MoCA, the Hong Kong version of Montreal Cognitive Assessment; MESOR, midline estimating statistic of rhythm; OR, odds ratio; CI, confidence interval.

[b] adjusted for age (years) and education (years), marital status, employment, diabetes, hypertension or cardiovascular diseases, sleep medication use, family history of dementia, smoking, alcohol drinking, tea drinker, coffee drinker, anxiety, depression, physical activity, poor sleep (PSQI>5), waist circumference, and handgrip strength.

	Community referents	Pneumoconiosis patients	*P*-value
Sample size	148	186	
Percent rhythm	18.7 (13.4, 23.6)	17.6 (13.6, 23.6)	0.660
Amplitude	139.1 (91.6, 193.8)	113.2 (79.1, 169.9)	0.011
MESOR	278.6 (218.1, 335.7)	236.4 (194.9, 309.5)	0.001
Acrophase	14.0 (12.5, 15.6)	13.5 (12.2, 14.8)	0.057

Supplementary Table S2. Comparisons of circadian activity rhythm between pneumoconiosis patients and community referents (exclude community subjects recruited during the COVID-19 pandemic)

Values were given as median (interquartile range).

Abbreviation: MESOR, midline estimating statistic of rhythm.
